# Comorbidity costs for the healthcare of mental patients

**DOI:** 10.1192/j.eurpsy.2024.650

**Published:** 2024-08-27

**Authors:** P. Dr. Fadgyas-Freyler

**Affiliations:** Health Policy, Corvinus University Budapest, Budapest, Hungary

## Abstract

**Introduction:**

Most patients with mental disorders exhibit multiple comorbidities. Without doubt the presence of multiple co-occurring somatic and mental disorders is associated with a higher insurance spending for the psychiatric patients. The details of this association need to be elucidated.

**Objectives:**

The aim of current study was 1) to delineate the typical nonmental comorbidities that occur among mental patients, and 2) to investigate social health insurance spending on comorbidities compared to the direct mental care costs of the same population. The analysis offers unique insight into the health care spending, since it focuses not only the costs of psychiatric care but reflects the whole range of treatments delivered to this group.

**Methods:**

A database with the claim records of the Hungarian NHIF was created including direct healthcare costs for mental diagnosis. Patients were recorded either in primary or in specialist care with at least one mental health diagnosis in the last pre-pandemic year (2019). Adopting a case-control design, spending and comorbidities were compared to the control group, which comprised patients who did not have any mental diagnosis. Cases and controls were matched on demographic characteristics like age, gender, place of residence with deprivation index and marital status..

**Results:**

Mental problems affected in 2019 more than 1,5 million persons in Hungary. Half of them did not access specialist care but were only seen with the mental diagnosis by a GP. Direct insurance spending for mental care is around 156 million EUR/year with 4% of the total direct health spending. Besides these costs another 665 million EUR (+17% of all health spending) were reimbursed for the same patient group for the treatment of other diseases. With regards to affected patient numbers, the three most important comorbidities were cardio-vascular conditions (34% of mental patients with 14% of all spending of the group); diseases of the digestive system (29% vs 14%); and musco-sceletal conditions (28% / 9% ) In terms of spending three other disease groups also have to be considered as of high significance: carcinomas (4% patients vs 13% of spending); neurological disorders (13% /vs 7%); and diseases of the endocrine, nutritional and metabolic system (24% vs 6%).

**Conclusions:**

The analysis aims to raise awareness for the complex issues of comorbidities of mental patients. We see that this patient group suffers heavily from other conditions the costs of which is much higher than the direct mental care costs. A better understanding of the coexistence of somatic and mental disorders and a holistic approach of treatment (care integration, reimbursement across different types of care, etc.) would be desirable.

**Disclosure of Interest:**

None Declared

